# A new record of the rare alga *Pachycladella* P. C. Silva (Chlorophyceae) in New England

**DOI:** 10.3897/phytokeys.56.6268

**Published:** 2015-09-15

**Authors:** Karolina Fučíková

**Affiliations:** 1 Department of Ecology and Evolutionary Biology, University of Connecticut, 75 North Eagleville Rd, Storrs, CT, USA

**Keywords:** Connecticut, floristics, microalgae, plankton

## Abstract

A rarely reported taxon, the microscopic green alga *Pachycladella*, was found in a pond in Connecticut. Due to an unresolved taxonomic debate within the genus, the species-level identity of the newly discovered population cannot be determined with absolute certainty. However, according to the currently accepted classification the Connecticut specimens best match *Pachycladella zatoriensis*, heretofore only known from Europe. The find represents not only the first record of *Pachycladella* in Connecticut, but also in the entire New England region. This study highlights the need for continuing floristic surveys even in regions previously well explored.

## Introduction

USA’s New England region, which encompasses the states of Connecticut (CT), Maine, Massachusetts, New Hampshire, Rhode Island and Vermont, has certainly received plenty of floristic attention over the past century. Freshwater algal floristic works in this region, however, have dwindled in recent years. In 1984, a 1020 pages long list of New England algal records and the related literature was compiled by Colt (1984) from previously published works, but relatively few studies have been added since. Yet, even those few have demonstrated the need for continuing floristic work on New England freshwater algae: for example, Vaccarino et al. (2011) added 140 generic records to the flora of Acadia National Park and Fučíková et al. (2015) reported 65 new species records for Maine. Clearly, much of the freshwater algal diversity still remains to be documented even in this relatively well-studied region.

The genus *Pachycladella* was originally described from Palisades Interstate Park in New York by Smith (1924, as *Pachycladon* G.M. Smith), and remained monotypic for nearly four decades. The type species, *Pachycladella umbrina* (G.M. Smith) P.C. Silva, is represented in public culture collections by a single strain (SAG 10.85 and its duplicates in other collections), which has been studied extensively in terms of its morphology, life cycle, and ultrastructure (e.g., Reymond and Hegewald 1990, Friedl and Reymond 1997). This strain was also used in molecular phylogenetic reconstructions. Both molecular data and ultrastructural features indicate that the strain (and presumably the genus) belongs to Chlorophyceae, and molecular phylogenies, e.g., in Škaloud et al. (2013), show it as member of the volvocalean clade Stephanosphaerinia.

*Pachycladella* and the species within it have a somewhat convoluted taxonomic history (Smith 1924, Silva 1970, Reymond 1980, Reymond et al. 1992). Of the five currently recognized species, three are not well understood in terms of morphological variation, occurrence, and ultimately taxonomic status (Reymond et al. 1992, Guiry and Guiry 2015). Through careful morphological examinations and literature review Reymond et al. (1992) clarified the blurry distinction between *Pachycladella umbrina* and *Pachycladella zatoriensis* (Bednarz & Mrozinska-Webb) Komárek, but the distinctness of *Pachycladella chodatii* (Bern.) Hegewald, *Pachycladella minor* (Chudybowa & Chudyba) P.C. Silva, and *Pachycladella komarekii* (Fott & Kovácik) Reymond still needs to be confirmed (Komárek and Fott 1983).

The present study contributes a new record of Pachycladella cf. zatoriensis from the USA, and is the first report of the genus *Pachycladella* for CT and for New England. Given the past taxonomic confusion it is difficult to interpret all historical records with certainty, but it is possible that this study also represents the first record of *Pachycladella zatoriensis* in the United States. Continuing survey studies will be necessary to understand the occurrence patterns of *Pachycladella* and its species.

## Methods

In 2014 and 2015 I conducted a survey of selected lakes, streams, and wetlands in northeastern CT and recorded the algal diversity in these habitats. Samples were collected using a 10 µm mesh plankton net, stored in small Ziploc bags, and examined microscopically using an Olympus BX60 microscope with Nomarski DIC optics equipped with an Olympus DP25 digital camera (Olympus Imaging America, Center Valley, PA, USA). The Olympus CellSens software was used to capture images and measure cell dimensions. Georeferenced records of observed algae, accompanied by micrographs where possible, have been deposited in iNaturalist (https://www.inaturalist.org/projects/freshwater-algae-of-new-england). Most algal species were identified using North American and New England taxonomic literature (Conn and Webster 1908, Hylander 1928, Prescott 1964, Whitford and Schumacher 1984).

## Results

**Figure 1.**
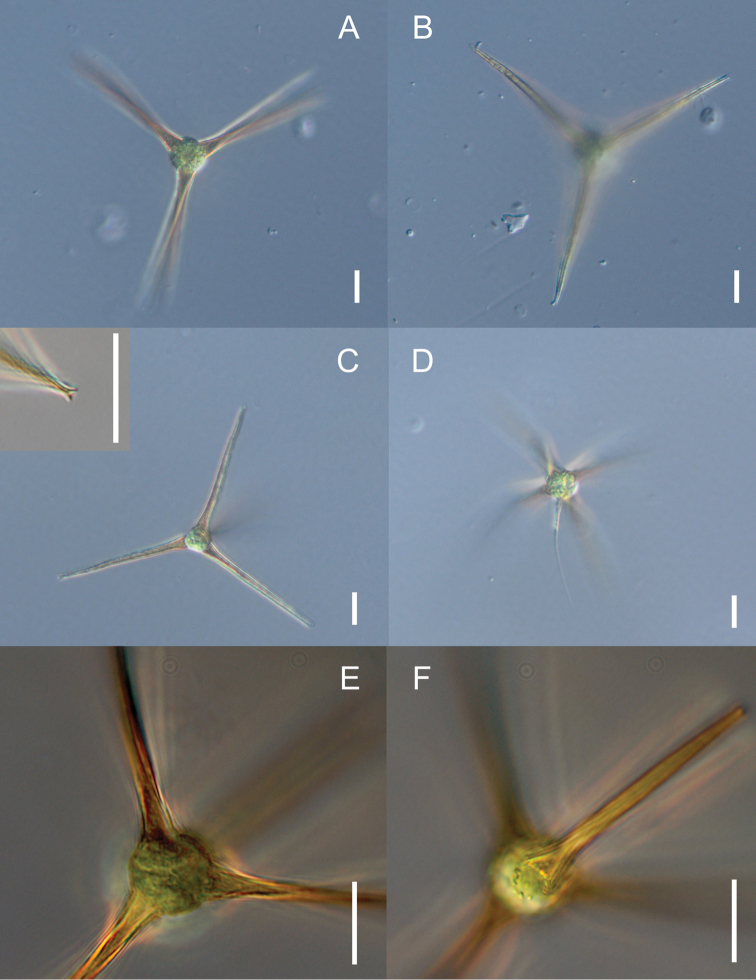
Light micrographs of Pachycladella cf. zatoriensis found in Connecticut. **A** gross morphology of a vegetative cell **B** same cell as in **A**, with focus on process apices **C** small vegetative cell with a clearly bifurcated process apex (enlarged in the inset) **D** an unusual cell with a fifth, irregularly placed and colorless appendage **E** high-magnification micrograph showing the hollow bases of cell wall processes as well as their dark coloration **F** same cell as in E with focus on the cell surface and attachment of the fourth process. Scale bars represent 10 µm in all images.

On August 4^th^ 2015, I collected a plankton sample from a pond on Bonemill Rd., Storrs, CT (41°48'12", -72°16'48") and in it found a population of a species I had not seen in any previous collection, including a 2014 sample from the same pond. I identified the alga as *Pachycladella umbrina* based on the unmistakable reddish-brown cell wall processes (Fig. 1A–F) that were either bluntly or bifurcately terminated (Fig. 1C). Upon later review of the literature it became apparent that the tetrahedral arrangement of the processes (also referred to as appendages in past literature) is characteristic of *Pachycladella zatoriensis* rather than *Pachycladella umbrina*, the latter of which has predominantly a cruciate arrangement of processes, i.e., all four processes are in the same plane (Reymond et al. 1992).

I collected an additional sample on August 12^th^. In both samples, *Pachycladella* occurred infrequently but consistently: I usually saw 5-10 specimens on every slide. Other algae abundant in the planktonic community included nonmotile colonial chlorophytes resembling *Chlamydocapsa planktonica* (West & G.S. West) Fott, members of the chlorophycean family Hydrodictyaceae, the large dinophyte *Ceratium hirundinella* (O.F. Müller) Dujardin, alongside diatoms (e.g., *Cymbella* C. Agardh, *Eunotia* Ehrenberg, and *Melosira* C. Agardh), synurophytes (*Mallomonas* Perty and *Synura* Ehrenberg), and euglenoids (*Euglena* Ehrenberg, *Lepocinclis* Perty, *Phacus* Dujardin, and *Trachelomonas* Ehrenberg).

**Figures S1.**
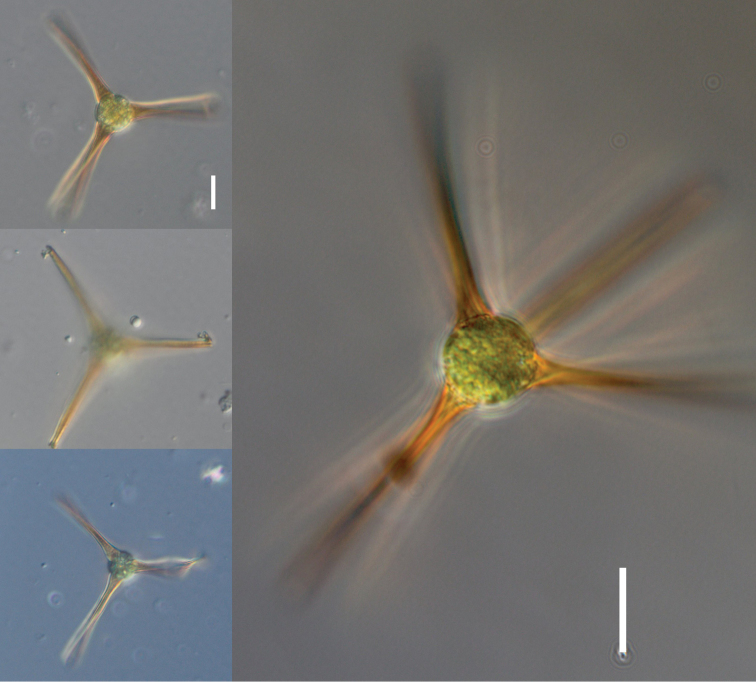
Additional light micrographs showing the morphology of Pachycladella cf. zatoriensis found in Connecticut. Scale bars represent 10 µm.

The observed *Pachycladella* cells were spherical and 8–11.5 µm in diameter, and possessed one chloroplast with a single pyrenoid (not always visible). Cell wall was protracted into four (rarely five, Fig. 1D) long, hollow processes arranged in a tetrahedral, exceptionally somewhat irregular manner. Of the ca. 30 specimens observed, none exhibited a cruciate arrangement of processes. The processes were light to dark reddish-brown in color, (26-) 30–42 µm long, 3.5–4.1 µm thick at the base and 1.6–2 µm thick ca. at half their length, and had a rough surface. Process apices were either bluntly pointed or bifurcated (Fig. 1C). Additional micrographs showing the observed morphological variation are available online as Fig. S1.

## Discussion

Globally, *Pachycladella* has been reported broadly but infrequently. For some time, the genus and its sole species were only known from North America (e.g., as noted by Bourrelly 1966). The geographically scattered records of the genus now also include several European countries, Argentina, Bangladesh, Brazil, India, Singapore, Taiwan, and possibly Australia (Reymond et al. 1992 and references within, Keppeler et al. 1999, Islam and Alfasane 2005, Pham et al. 2011, Hentschke and Prado 2012). *Pachycladella umbrina* is by far the most commonly reported species, although many reports do not contain enough detail in morphological descriptions or figures to critically evaluate the species-level identifications. Thus, some of these reports may in fact represent the occurrence of another species of *Pachycladella*.

Based on past records and newly collected data, Reymond et al. (1992) argued that the tetrahedral vs. cruciate arrangement of processes is a stable trait within populations and can be used to distinguish *Pachycladella umbrina* (cruciate) from *Pachycladella zatoriensis* (tetrahedral). Following this distinction they assigned several previous records of *Pachycladella* to either of the two species, but also left many as uncertain due to lack of information in the reports or because the reported morphological variation spanned both types of appendage arrangement. *Pachycladella zatoriensis* is so far only confirmed to occur in Europe (Reymond et al. 1992). However, Reymond et al. (1992) also suggested that Playfair’s (1918) report of *Bernardia tetraedrica* from Australia is in fact the first report of *Pachycladella zatoriensis*. Notably, Playfair (1918) does not mention two of the generic characters assigned to *Pachycladella* by Smith (1924): a common bifurcation of the process apices and the brown tint of the cell wall. Both traits were also extensively documented in *Pachycladella zatoriensis* (Bednarz and Mrozinska-Webb 1971, Reymond et al. 1992, 1993). While these characters may be variable and facultative (Wawrik 1977, Reymond et al. 1993), their complete absence in Playfair’s description and figure makes the synonymy with *Pachycladella* far less certain.

In the USA, *Pachycladella* has been heretofore reported from Alabama, Kentucky, New York, North Carolina, Tennessee, and from the Great Lakes region, and has been noted to be rare or uncommon (e.g., Prescott 1964, Shubert 2003). The genus was not found during the extensive National Lakes Assessment conducted by the United States Environmental Protection Agency in 2007. Only *Pachycladella umbrina* has been reported thus far, but many US records of *Pachycladella* cannot be assigned to species with confidence (Reymond et al. 1992 and references within).

The population of *Pachycladella* newly found in CT morphologically matched the original description of *Pachycladella umbrina* except for having appendages consistently in a tetrahedral orientation, rather than being mostly cruciate (Smith 1924). The tetrahedral disposition classifies the CT specimens as *Pachycladella zatoriensis* *sensu* Reymond et al. (1992), which has heretofore not been reported from the USA. The original description of *Pachycladella zatoriensis* by Bednarz and Mrozinska-Webb (1971) however reports slightly smaller cells and considerably shorter processes, only up to 20 µm long. The size difference between the two species appears to be consistent across the reports reviewed and assigned to species by Reymond et al. (1992). With their larger size and tetrahedral appendage orientation, the CT specimens fit neither species perfectly, nor do they fit other *Pachycladella* species morphologically or ecologically. However, several reports marked as uncertain by Reymond et al. (1992) suggest a similar trait combination as the CT specimens (e.g., Wawrik 1977, Dillard 1989). Thus, the distinction between the two species may not be as clear-cut as implied by Reymond et al. (1992).

The rarity of *Pachycladella* combined with difficult-to-interpret past records makes species-level taxonomy in this genus quite tricky. DNA barcode data are available only from SAG 10.85, and therefore genetic comparisons of morphologically and geographically distinct populations are not possible at present. A morphological study aided by a molecular phylogeny would help deciding which traits should be considered taxonomically informative. Because even morphologically distinct taxa can be non-monophyletic (e.g., McManus and Lewis 2011), such a study on *Pachycladella* would also elucidate whether the *Pachycladella*-like morphology has a common origin or represents multiple taxa. For the purposes of this study, I followed the taxonomic scheme of Reymond et al. (1992) and assigned the CT specimens to *Pachycladella zatoriensis*. However, it is possible that they represent an unusual population of *Pachycladella umbrina*, or possibly even a novel species of *Pachycladella*. Given this uncertainty I am hesitant to proclaim this find the first US record of *Pachycladella zatoriensis*, but based on the available literature it is the first report of the genus and species in New England.

It is possible that this interesting alga had been overlooked in past floristic studies or misidentified as another taxon, despite its distinct morphology. *Pachycladella* could conceivably be mistaken for *Treubaria triappendiculata* Bernard, a species reported from Massachusetts by Gustafson (1942). It could also possibly be confused with other species of *Treubaria* (which have been reported from Maine and New Hampshire, Colt 1984 and references within). However, the two genera, despite both bearing tetrahedrally or cruciately arranged processes, are quite morphologically different. When present, the brown cell wall and bifurcate processes give *Pachycladella* away immediately. Furthermore, e.g., Smith (1950) summarizes the differences in cell shape (spherical in *Pachycladella* vs. pyramidal in *Treubaria*), appendage morphology (slender, brown and blunt/bifurcate in *Pachycladella* vs. stout, hyaline, and sharp in *Treubaria*), and chloroplast features (single plastid with one pyrenoid in *Pachycladella* vs. four pyrenoids or even four distinct plastids in older cells of *Treubaria*). Other than the unlikely misidentification for *Treubaria*, no records of *Pachycladella* or its synonyms are listed in Colt’s New England checklist (Colt 1984) or subsequent works (e.g., Sheath and Harlin 1988, Vaccarino et al. 2011).

It may be somewhat surprising to see a new genus record in a region so well studied – and particularly in CT, which of all the New England states likely has the best documented freshwater algal microflora, perhaps only rivaled by Rhode Island (Conn and Webster 1908, Hylander 1928, Colt 1984, Sheath and Harlin 1988). However, many of the comprehensive studies are several to many decades old, and given the drastic environmental and habitat changes this region has undergone in the past century, one cannot simply assume that the same biodiversity still occurs here. As shown in the present study, a previously unreported taxon may have expanded its range into New England from more southern states. In other cases, species may have been lost from New England due to habitat deterioration or destruction without this biodiversity loss ever being noticed. With this small study I hope to underscore the importance of continuing survey studies on algae, as they are key players in aquatic ecosystems.

## Conclusion

Despite past taxonomic confusion and uncertainty about the classification and specific diversity of *Pachycladella*, this study presents a new record of an alga that matches the original generic description perfectly, best matching the species *Pachycladella zatoriensis*. As such, it is the first record of *Pachycladella* in New England and possibly the first record of *Pachycladella zatoriensis* in North America.
